# Supplementary material for: Exome and genome sequencing of nasopharynx cancer identifies NF-κB pathway activating mutations
Source: Nat Commun. 2017 Jan 18;8:14121. doi: 10.1038/ncomms14121 (PMC5253631; doi:10.1038/ncomms14121)
Supplement: Supplementary Information — Supplementary Figures 1-15 and Supplementary Table 1 [file ncomms14121-s1.pdf]

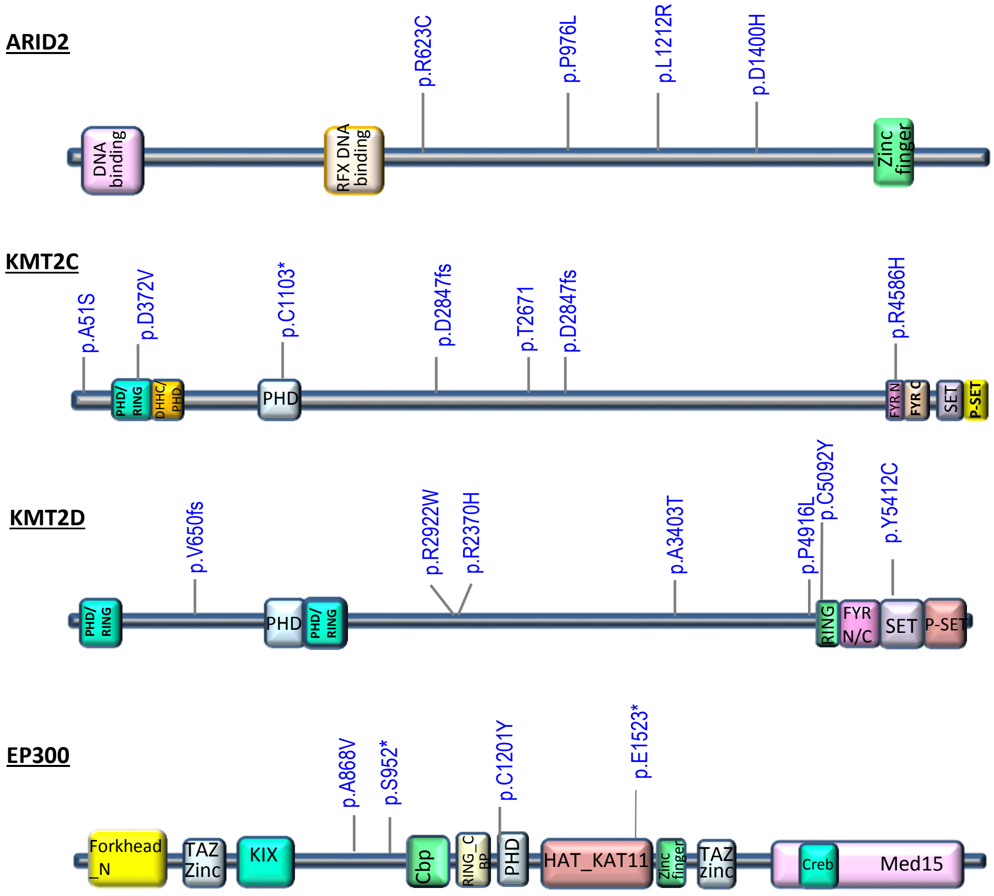

Supplementary Figure 1. Mapping of mutation sites in *ARID2, KMT2C, KMT2D*, and *EP300* based on the NPC WES data. The functional domains of all genes are defined as in the UniProtKB/SwissProt database.

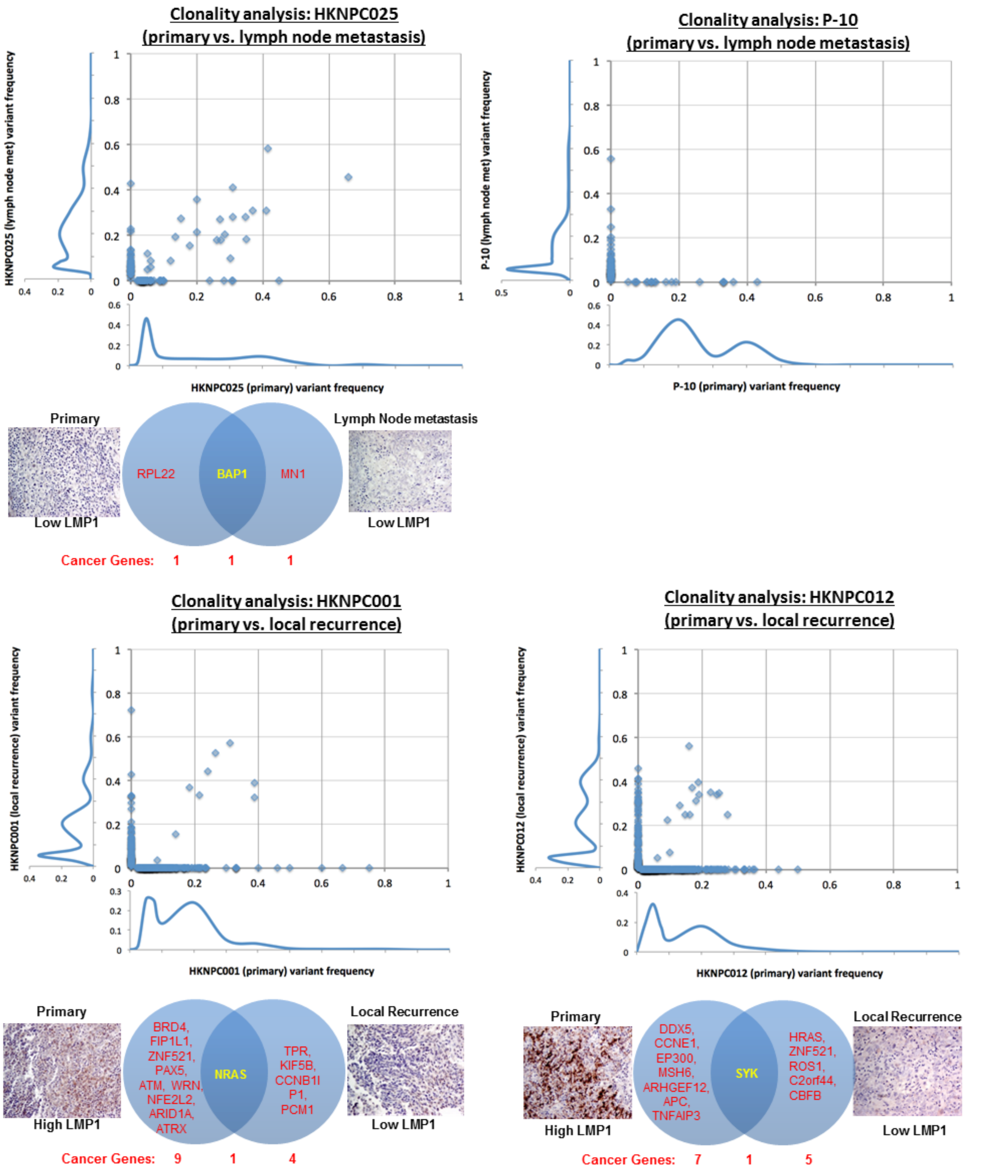

Supplementary Figure 2. Mutation clonality plots. Plots for paired primary/recurrent NPC cases (HKNPC001-primary/local recurrence, HKNPC012-primary/local recurrence) or primary/metastatic NPC cases (HKNPC025 and P-10, respective primary/lymph node metastasis). Each upper panel: variant frequency of mutations in each patient and distribution of mutations at each variant frequency, identified by WES. Each lower panel: number of cancer genes (COSMIC cancer gene census) that

were identified solely in the primary or in the recurrent/metastatic tumors (red), and the genes that were shared between these (yellow). The respective LMP1 staining of the specimens are also shown. For HKNPC001, the local recurrence again appeared to have more subclonal peaks compared to the primary. For HKNPC025, the lymph node metastasis demonstrated the emergence of new low-frequency subclones. In contrast, P-10 appeared to be a unique case, with no shared mutation between the lymph node metastasis and the primary, as well as completely differing clonal landscapes, suggesting the two tumors did not arise from a single tumor origin. Mutation data for each pair are shown in the Supplementary Dataset.

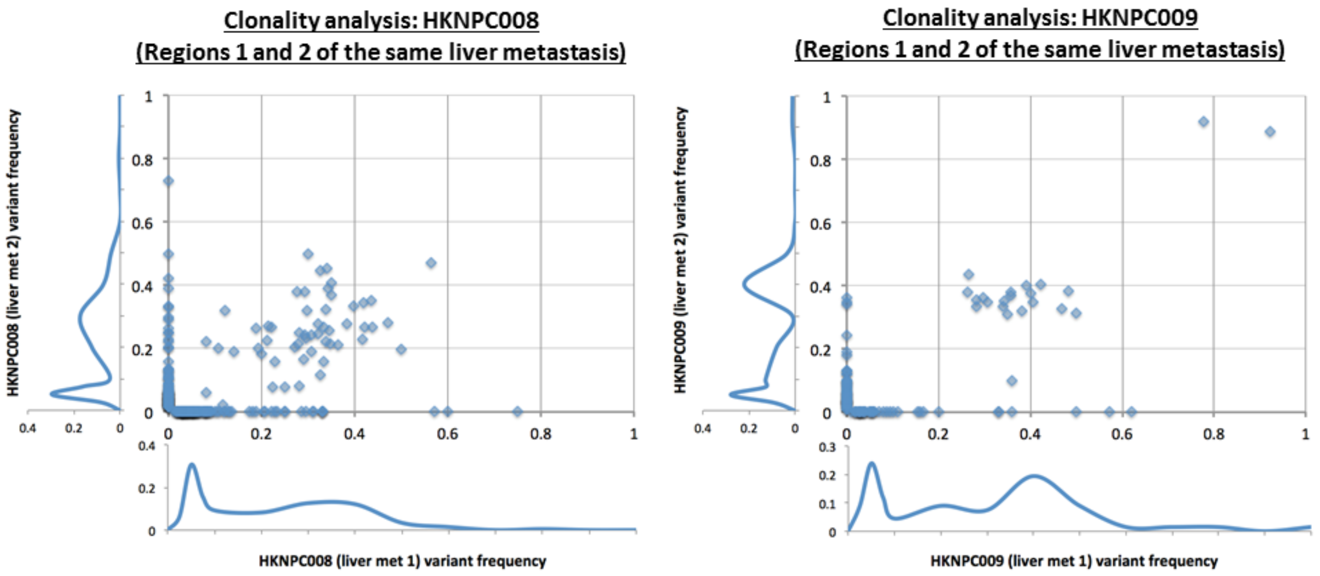

Supplementary Figure 3. Tumor heterogeneity of NPC liver metastases as revealed by WES. The upper panel displays the variant frequency identified in 2 different regions (1 & 2) of the same liver metastasis of patient HKNPC008. The two liver metastases of HKNPC008 and HKNPC009 both showed a higher percentage of shared mutations within each patient's tumors. Mutation data for each pair are shown in the Supplementary Dataset.

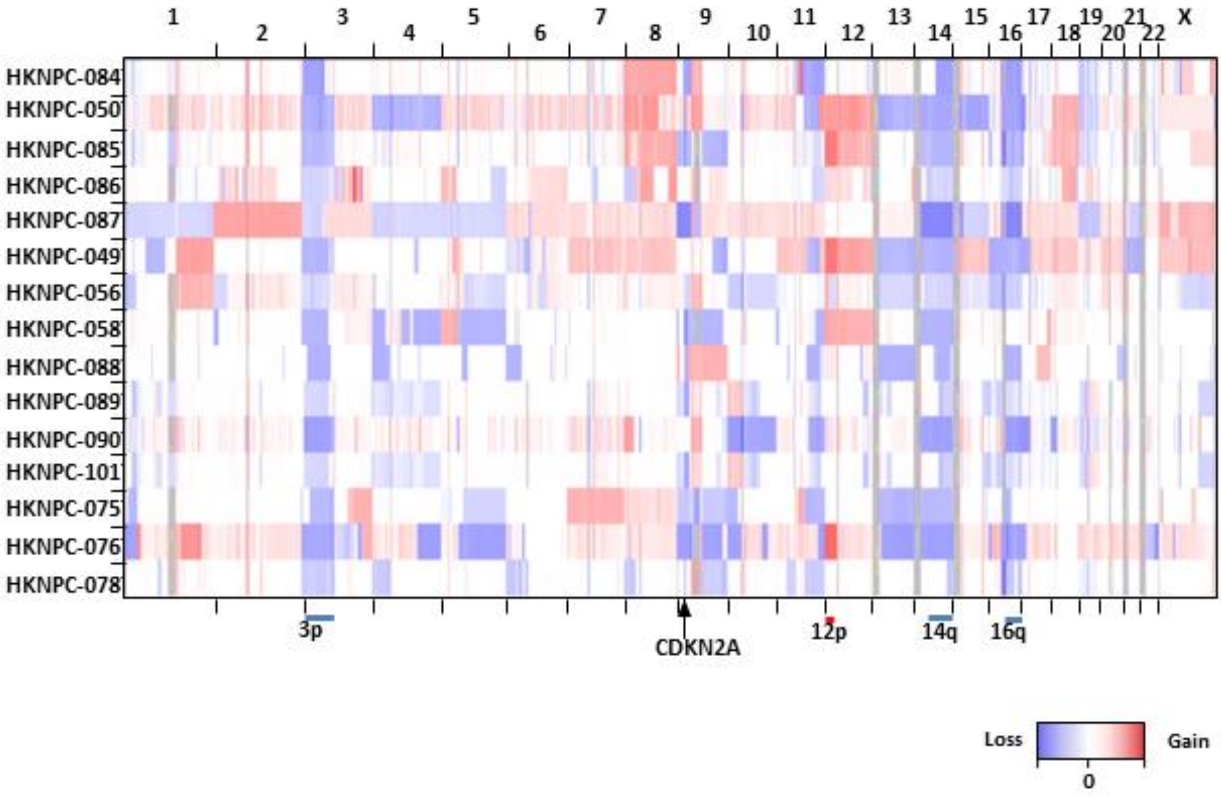

Supplementary Figure 4. Heat map representation of gains and losses in the genomes of 15 NPC tumors. Consistent loss of 3p, 14q and 16q and recurrent amplification of 12p are shown. Blue color indicates losses, and red color depicts gains.

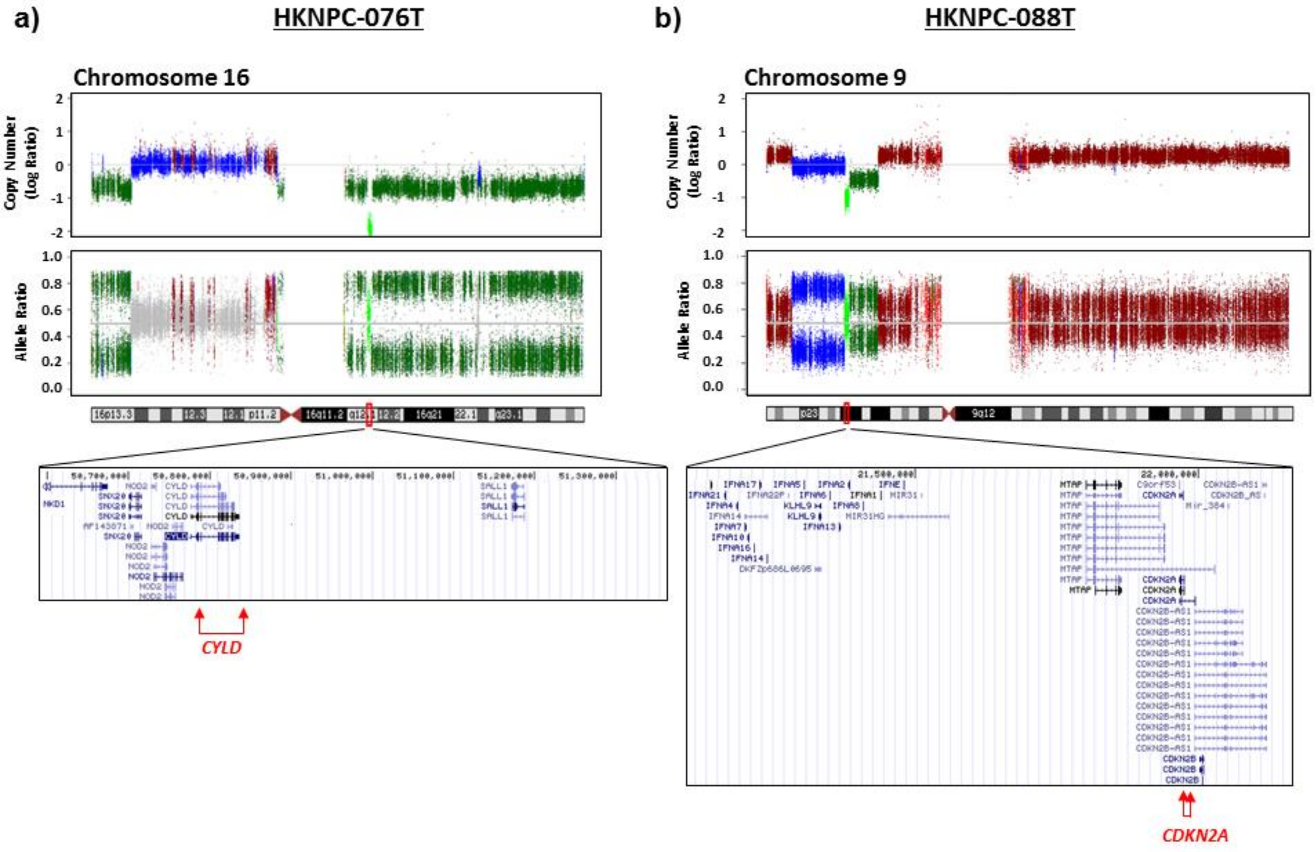

Supplementary Figure 5. Copy number and allele ratio analysis revealed homozygous deletion of *CYLD* and *CDKN2A* in representative NPC cases. a) In case HKNPC076T, the copy number and allele ratio plots of chromosome 16 show a homozygous deletion region at 16q12.1 (Chr16: 50593084-51362792). The 770kb region harbors five genes including *CYLD, NOD2, SNX20, NKD1 and SALL1.* b) A homozygous deletion region at 9p21.3 (Chr9: 21,094,833-22,205,243) was also detected in HKNPC-88T. The region contains the *CDKN2A* gene which is frequently inactivated in NPC. For copy number plots: copy neutral, deletion, amplification are represented by blue, green, and red, respectively. For allelic ratio plots: LOH, copy neutral LOH (NLOH), diploid heterozygous (HET), and allele-specific amplification (ASCNA) are shown as green, blue, grey, red, respectively. Homozygous deletion is indicated by light green.

Supplementary Figure 6

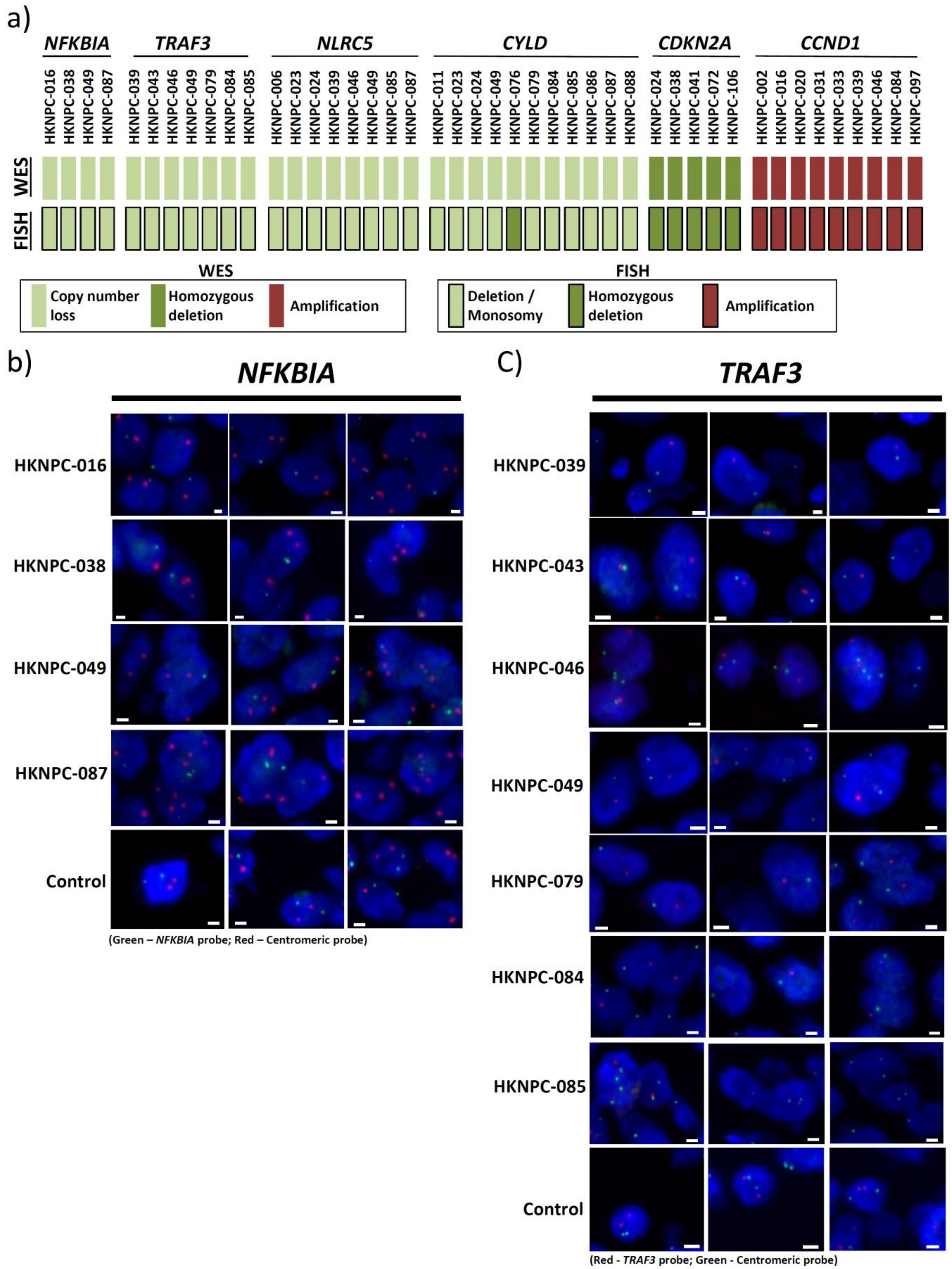

Supplementary Figure 6 (Cont'd)

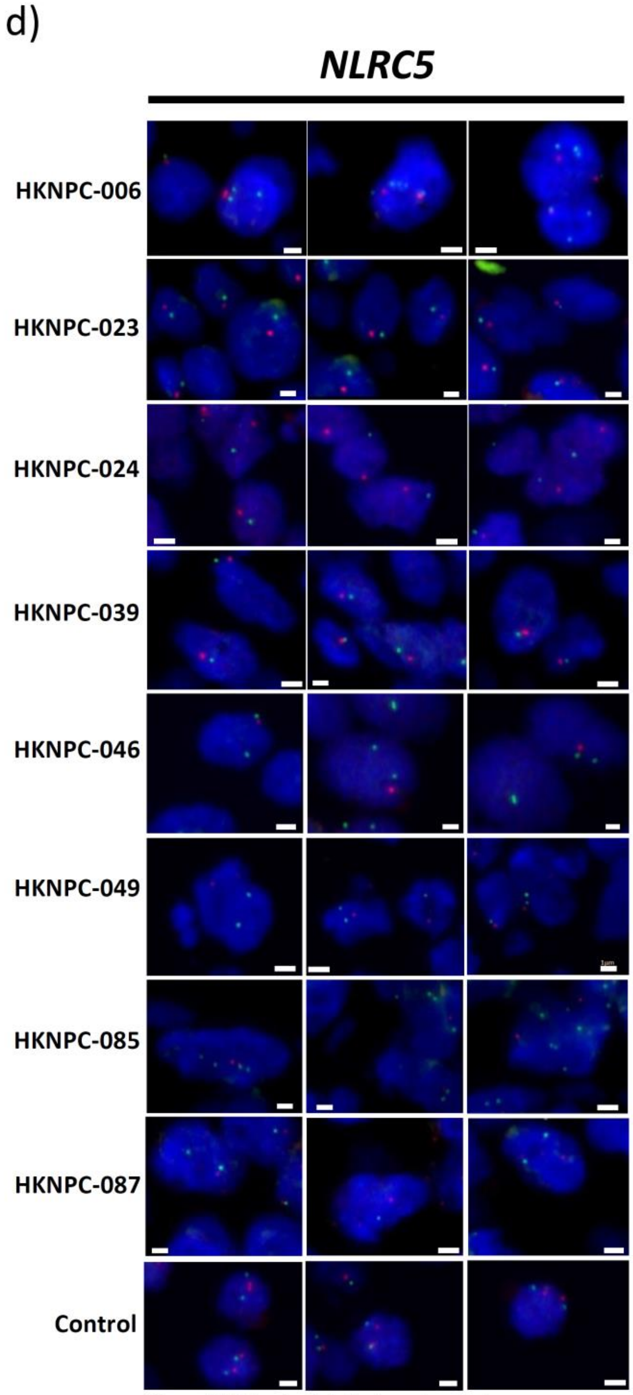

(Green – *NLRC5* probe; Red - Centromeric probe)

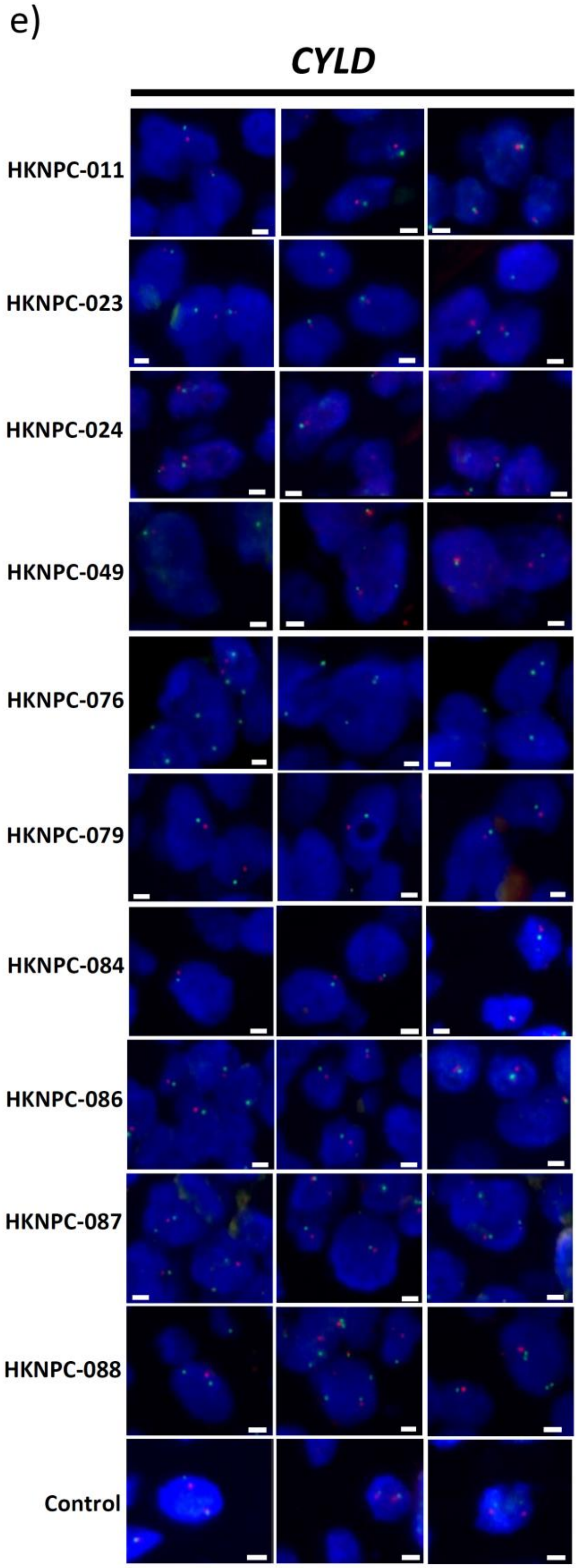

(Green – *CYLD* probe; Red - Centromeric probe)

Supplementary Figure 6 (Cont'd)

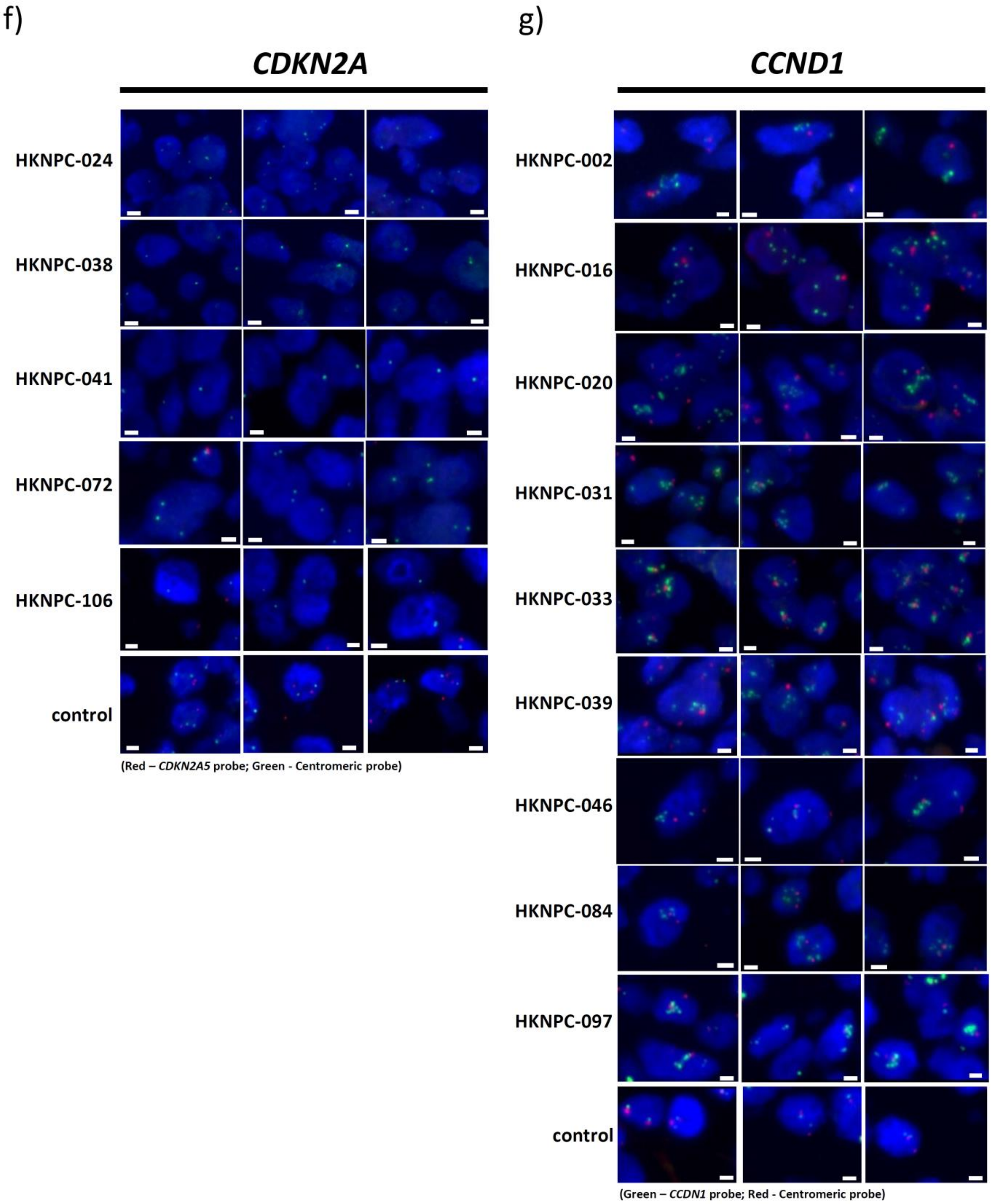

Supplementary Figure 6. Validation of CNVs identified in NPC by FISH analysis. a) A total of 44 CNVs of *NFKBIA, TRAF3, NLRC5, CYLD, CDKN2A* and *CCND1* genes in NPC cases were selected

for validation by FISH analysis. The CNVs examined include Copy number loss, homozygous deletion and amplification. For FISH analysis, chromosomal centromeric and gene specific probes were labelled as used for determining copy number changes. For each cases, the gene specific and control signals were scored in more than 100 tumor cells. Deletion was scored when the ratio of total gene specific signals and controls signals were <0.7. Monosomy was defined when both average gene specific and centrometric signals in each tumor cell was ≤ 1.2. In the 30 cases in which copy number loss was identified by WGS, 29 cases showed either deletion or monosomy in FISH analysis. For the case HKNPC-076, homozygous deletion of *CYLD* was detected by FISH analysis while WES data only showed copy number loss. In the selected NPC cases, CDKN2A homozygous deletion and *CCND1* amplification were detected by both WES and FISH analysis. Representative FISH figures indicating the CNVs of b) *NFKBIA*, c) *TRAF3*, d) *NLRC5*, e) *CYLD*, f) *CDKN2A*, or g) *CCND1* in each NPC was shown. Normal lymphocytes without CNVs were also included as controls. A scale bar (1μm) was included in all FISH figures.

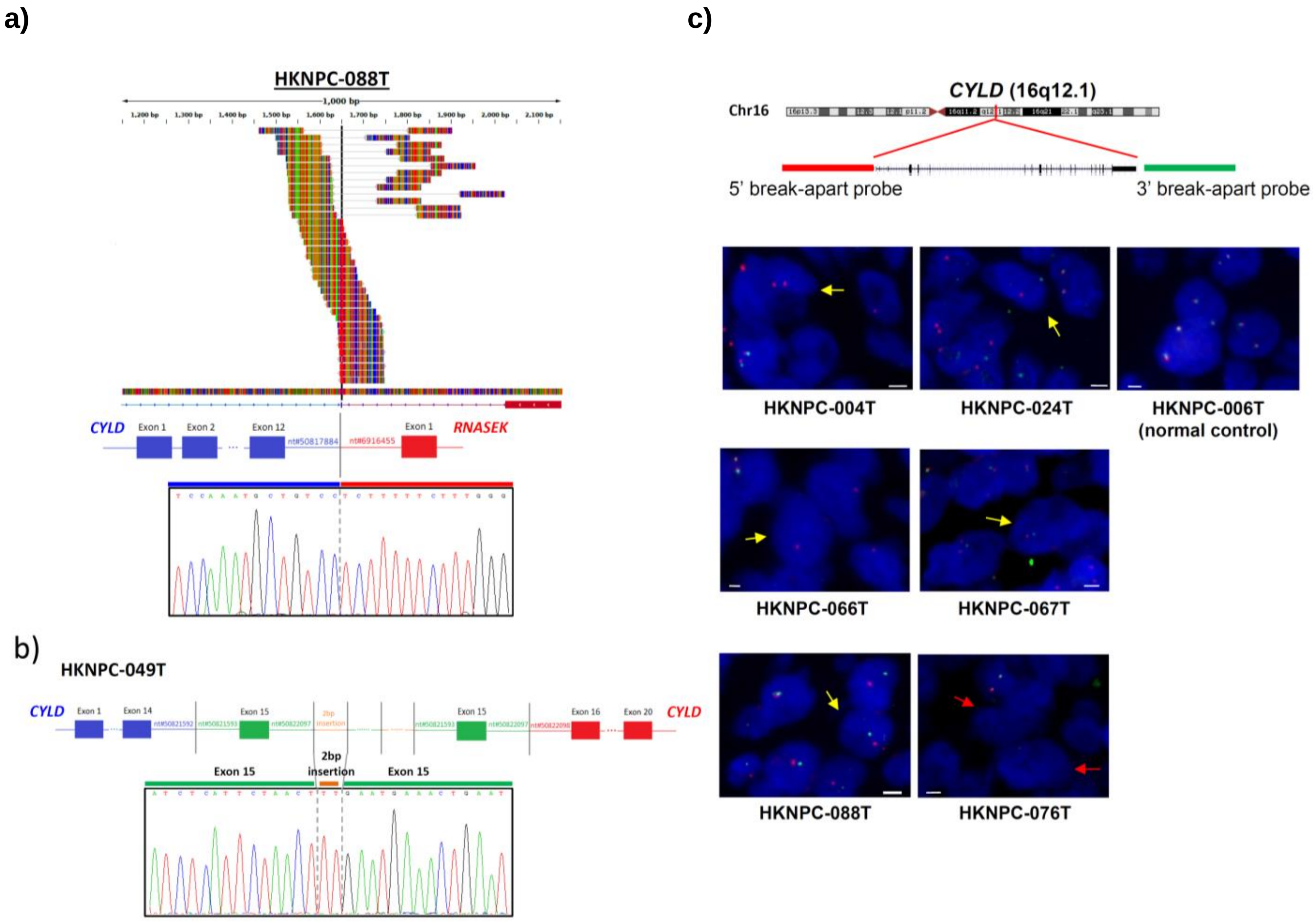

Supplementary Figure 7. Structural variations of *CYLD* in NPC detected by WGS and FISH analysis. a) The reads spanning the breakpoint junction of *CYLD* and *RNASEK* fusion in HKNPC-088T are illustrated using the Integrative Genomics Viewer (IGV). The fusion junction of *CYLD* intron 12 and *RNASEK* intron 1 was confirmed by Sanger sequencing. b) In HKNPC-049T, the predicated tandem duplication of *CYLD* was validated by Sanger sequencing. c) Using break-apart probes, *CYLD* gene rearrangement was detected in 4 additional tumor cases (HKNPC-004T, HKNPC-024T, HKNPC-066T, HKNPC-067T) by FISH analysis. Yellow arrow indicates the tumor cell with break-apart signals. HKNPC-076T with homozygous deletion of *CYLD* shows loss of both green and red signals in the tumor cells (Red Arrow). A scale bar (1μm) was included in all FISH figures.

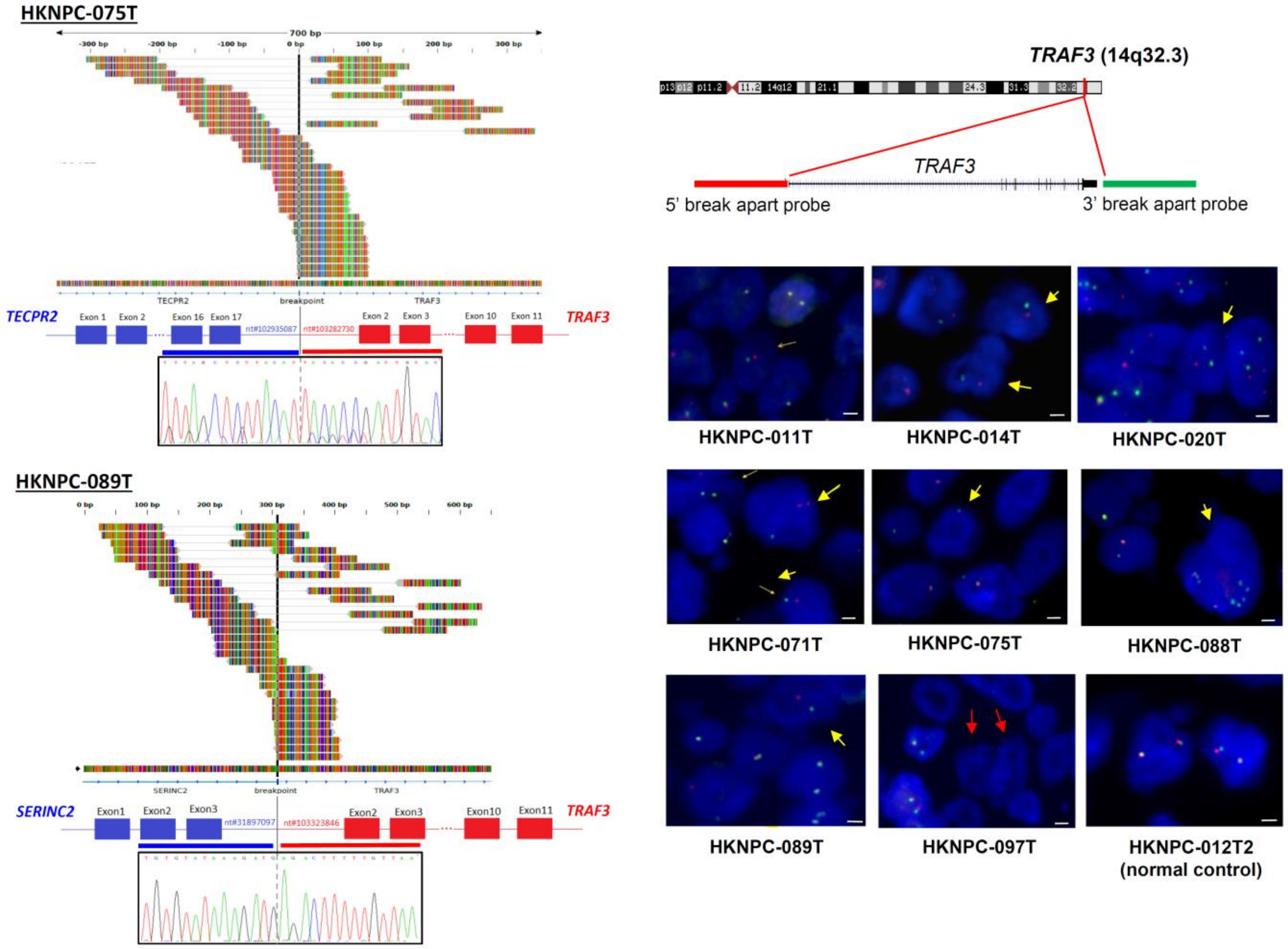

Supplementary Figure 8. Structural variations of *TRAF3* in NPC. SVs of *TRAF3* detected by WGS and FISH analysis were illustrated. In HKNPC-075T and HKNPC-089T, IGV view shows the reads spanning the junction breakpoints of *TRAF3-TECPR2* and *TRAF3-SERINC2* fusions, respectively. The *TRAF3* rearrangements of the cases were validated by Sanger sequencing and FISH analysis using break-apart probes. FISH analysis detected five additional NPC tumors with *TRAF3* gene rearrangements (HKNPC-011T, HKNPC-014T, HKNPC-020T, HKNPC-071T, and HKNPC-089T). Yellow arrows indicate the tumor cells with representative break-apart signals. Homozygous deletion was found in HKNPC-097T. Red arrows show the tumor cells with complete loss of green and red signals. A scale bar (1μm) was included in all FISH figures.

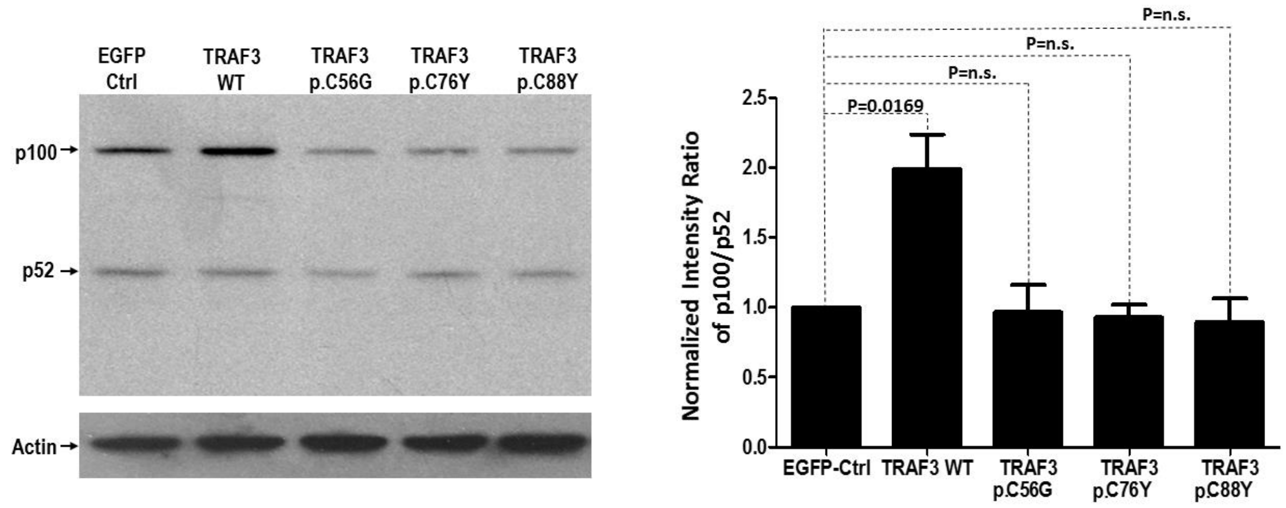

Supplementary Figure 9. *TRAF3* wildtype, but not *TRAF3* mutants inhibits p100 processing in NPC cells. HK1 cells were transiently transfected with *EGFP-Ctrl*, *TRAF3* WT, and *TRAF3* mutants (p.C56G, p.C76Y and p.C88Y). A representative Western blot result and a cumulative plot of quantification of p100 /p52 levels from three independent experiments were shown. *TRAF3* WT expression resulted in an increase in p100/p52 ratio (indicative of less p100 processing), while all *TRAF3* mutants displayed loss-of-function activities as compared to the *TRAF3* WT.

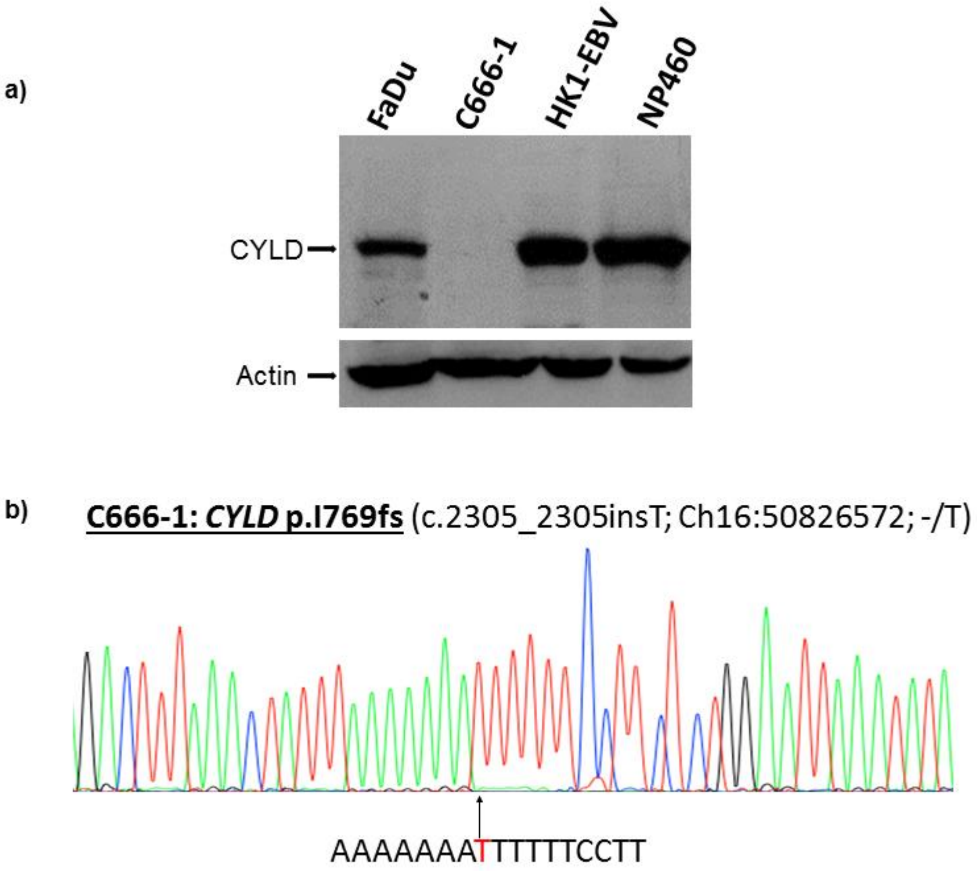

Supplementary Figure 10. *CYLD* p.I769fs mutation resulting in loss of CYLD protein in an NPC cell line, C-6661. a) Western blotting showing the absence of CYLD protein expression in C666-1 (an EBV-positive NPC cell line carrying endogenous *CYLD* p.I769fs mutation) as compared to FaDu (a control HNSCC cell line with *CYLD* WT gene) and HK1-EBV and an nasopharyngeal epithelial cell line, NP460. Actin was shown as loading control. b) Validation of *CYLD* mutation in C666-1 by Sanger sequencing.

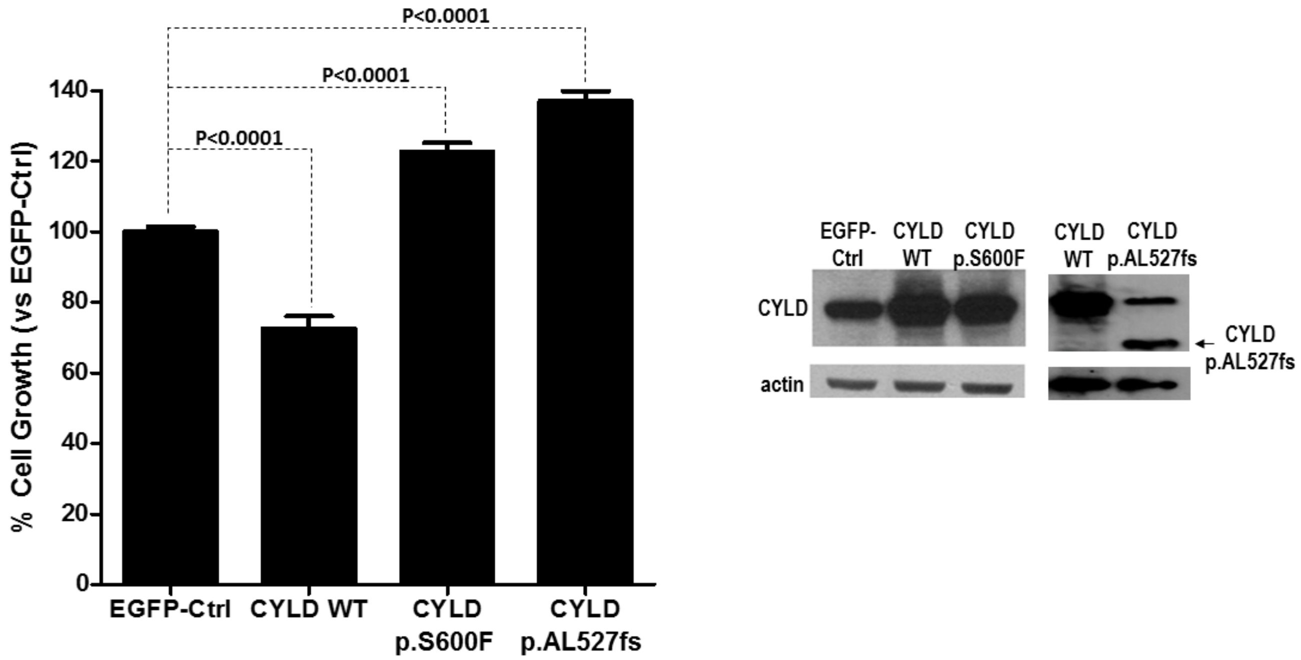

Supplementary Figure 11. *CYLD* wildtype (WT) gene suppressed the growth of HK1-EBV cells, while CYLD mutant lost such a growth-suppressive activity. HK1-EBV cells were infected with *CYLD* WT or mutants by retroviral infection. Cells were plated for 48h in 5% FBS, 1 x 10^4 cells, and then subjected to MTT assay. The subpanels showed the expression of the corresponding mutant CYLD protein bands as detected by Western blotting. Graph showing cumulative results from four independent experiments with four replicate wells each (total N=16).

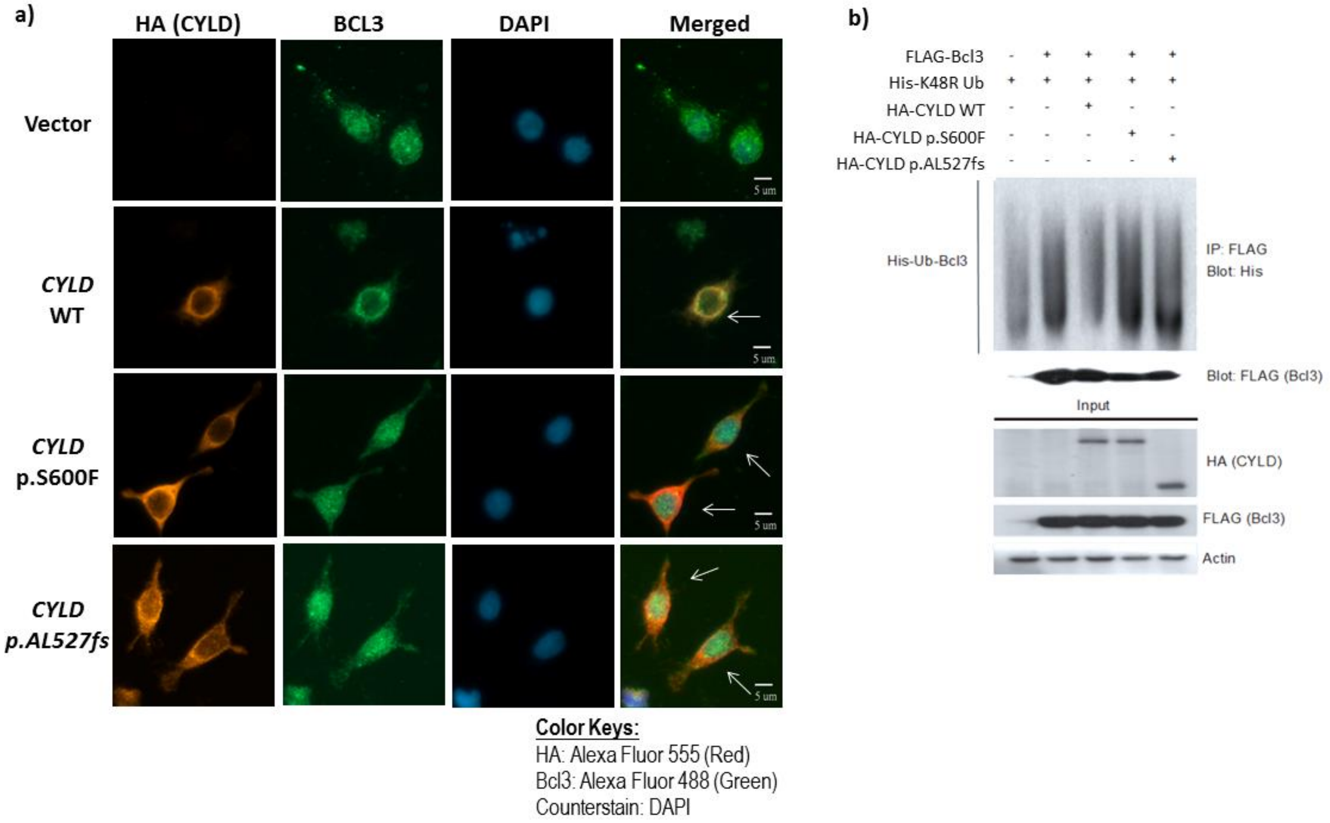

Supplementary Figure 12. Wild type *CYLD* inhibited nuclear translocation of BCL3 in NPC cells. By immunofluorescence assay, inhibition of nuclear accumulation of BCL3 (green) was demonstrated in the C666-1 cells expressing wild type *CYLD* (Red), but not in cells expressing the *CYLD* mutants (p.S600F and p.AL527fs) nor vector control. Arrows indicate the cells with wild type or mutated CYLD expression. A scale bar (5 µm) is shown. b) Demonstration of loss of deubiquination activity of CYLD mutants (p.S600F and p.AL527fs) on Bcl3. *CYLD* WT, as expected, deubiquitinated poly-ubiquitin chain of Bcl3. HeLa cells were co-transfected with the Flag-tagged Bcl3, the His-tagged K48R ubiquitin and together with *CYLD* WT or mutant-expressing plasmids. Cells were harvested at 24 hours post-transfection and ubiquitination assay was performed. Anti-His antibody was used to probe for the poly-ubiquitin chain of Bcl3. Protein expression of different plasmid constructs was shown as input in the figure. CYLD mutants were defective in deubiquitinating Bcl3 poly-ubiquitin chain.

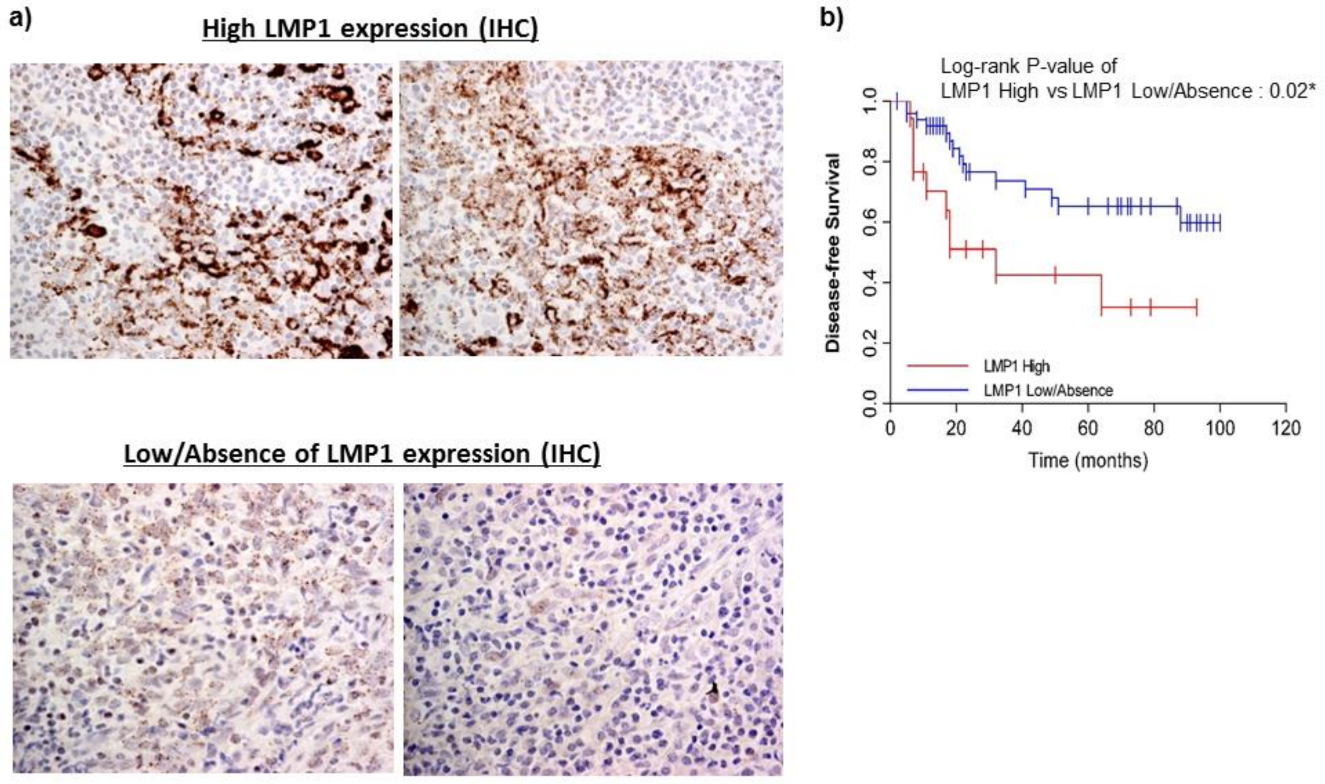

Supplementary Figure 13. Clinical correlation between LMP1 expression and disease-free survival in 70 primary NPC. High LMP1 expression is significantly associated with poorer disease-free survival (P=0.02; Log-rank test).

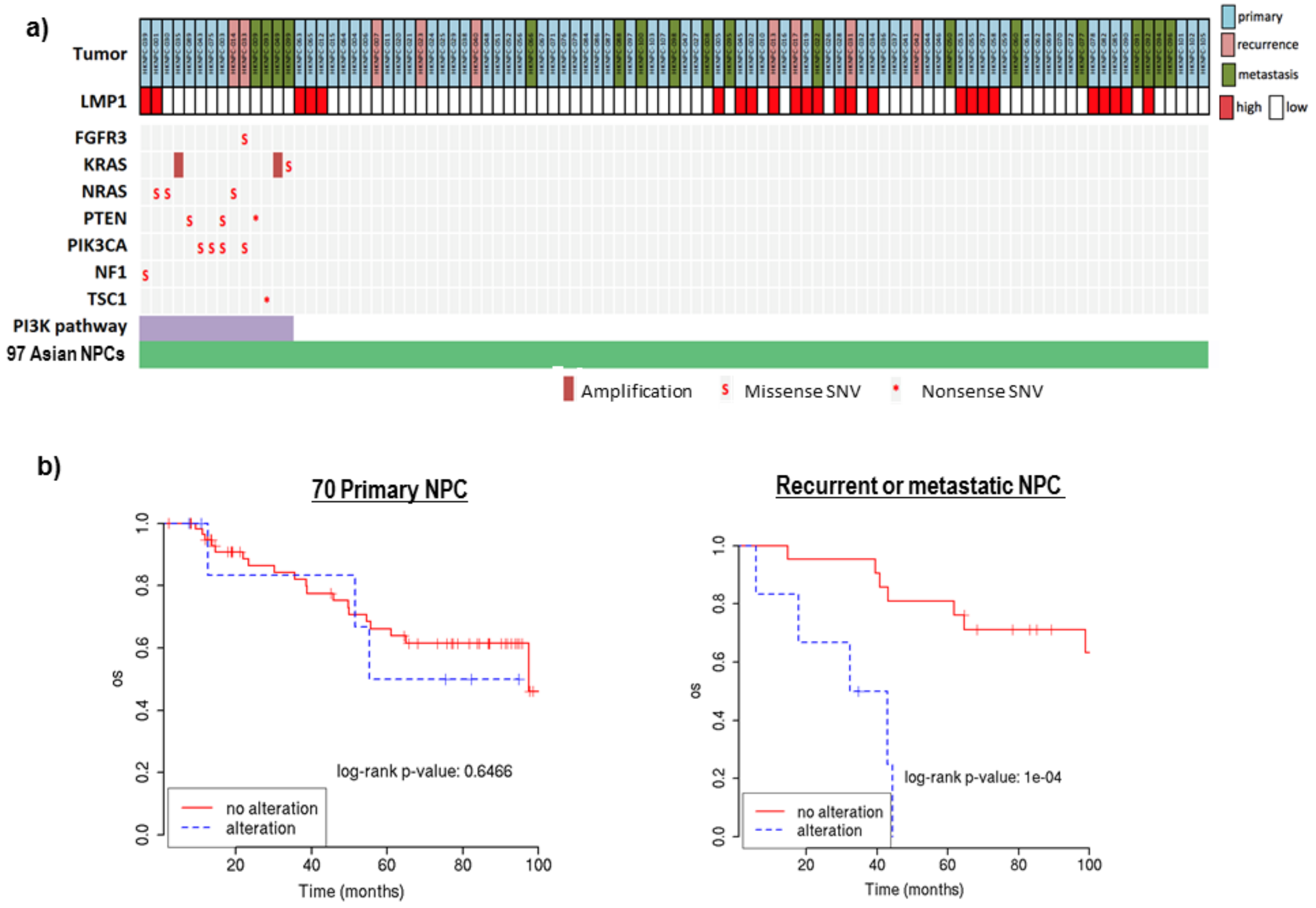

Supplementary Figure 14. PI3K activating somatic events in NPC. a) Pattern of PI3K activating somatic events in Asian NPC. b) Left: No association between PI3K somatic events and primary NPC in our Asian cohort (N=97). Right: Significant association among PI3K activating events and poor outcome in recurrent/metastatic Asian NPC. Amplification and mutation of multiple components of PI3K pathways are shown.

Figure 5C

CYLD

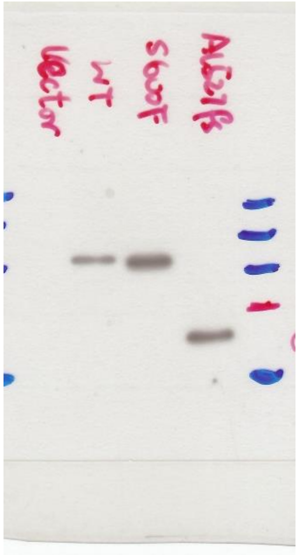

Figure 5C

actin

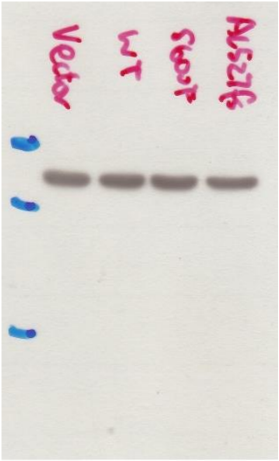

Supplementary Figure 15. Uncropped images of Western Blot membranes. Images corresponding to experiments presented in Figure 5C were shown.

Supplementary Table 1. Clinicopathological characteristics of our NPC cohort (N=105 individuals).

Characteristics	NPC Patients (n=105)	
**Age, years**		
Median (range)[#]	49	(23-85)
**Sex***		
Men	75	71.4%
Women	29	27.6%
**Ethnicity**		
Asian	97	92.4%
Caucasian	7	6.7%
N.A.	1	1.0%
**Tumor Type**		
Primary	78	74.3%
Recurrent	9	8.6%
Metastasis	18	17.1%
**EBER Status**		
Positive	105	100.0%
Negative	0	0.0%
**Disease Stage**		
I	6	5.7%
II	21	20.0%
III	43	41.0%
IV	31	29.5%
N.A.	4	3.8%
**Tumor Site**		
Nasopharynx	87	82.9%
Liver metastasis	6	5.7%
Lymph Node metastasis	10	9.5%
Lung metastasis	2	1.9%
**Tumor Grade***		
Non-keratinizing	100	95.2%
Keratinizing	4	3.8%

N.A. for 2 cases

* N.A. for 1 case
